# Tactical Scientific Decision-Making during Crewed Astrobiology Mars Missions

**DOI:** 10.1089/ast.2018.1837

**Published:** 2019-03-06

**Authors:** A.H. Stevens, S.E. Kobs Nawotniak, W.B. Garry, S.J. Payler, A.L. Brady, M.J. Miller, K.H. Beaton, C.S. Cockell, D.S.S. Lim

**Affiliations:** ^1^ UK Centre for Astrobiology, School of Physics and Astronomy, University of Edinburgh, Edinburgh, UK.; ^2^ Department of Geosciences, Idaho State University, Pocatello, Idaho, USA.; ^3^ NASA Goddard Space Flight Center, Greenbelt, Maryland, USA.; ^4^ School of Geography and Earth Sciences, McMaster University, Hamilton, Canada.; ^5^ Georgia Institute of Technology College of Engineering, Atlanta, Georgia, USA.; ^6^ NASA Johnson Space Center, Houston, Texas, USA.; ^7^ NASA Ames Research Center, Moffett Field, California, USA.

**Keywords:** Decision-making, Mars, Science operations, EVA, Tactical

## Abstract

The limitations placed upon human explorers on the surface of Mars will necessitate a methodology for scientific exploration that is different from standard approaches to terrestrial fieldwork and prior crewed exploration of the Moon. In particular, the data transmission limitations and communication latency between Earth and Mars create a unique situation for surface crew in contact with a terrestrial science team. The BASALT research program simulated a series of extravehicular activities (EVAs) in Mars analog terrains under various Mars-relevant bandwidth and latency conditions to investigate how best to approach this problem. Here we discuss tactical decision-making under these conditions, that is, how the crew on Mars interacts with a team of scientists and support personnel on Earth to collect samples of maximum scientific interest. We describe the strategies, protocols, and tools tested in BASALT EVAs and give recommendations on how best to conduct human exploration of Mars with support from Earth-based scientists. We find that even with scientists supporting them, the crew performing the exploration must be trained in the appropriate scientific disciplines in order to provide the terrestrial scientists with enough information to make decisions, but that with appropriate planning and structure, and tools such as a “dynamic leaderboard,” terrestrial scientists can add scientific value to an EVA, even under Mars communication latency.

## 1. Introduction

NASA currently has plans to launch a crewed mission to Mars in the coming decades (Craig *et al.,* 2015), with the goal of *in situ* scientific analysis leading into sample return. Unlike during the Apollo missions, when astronauts sent to the surface of the Moon were rarely scientists by training (El-Baz, 2011; Lofgren *et al.,* 2011), the crews of future Mars missions are likely to include astronauts with professional backgrounds or significant experience in relevant sciences such as geology and biology, both critical fields in the search for life on other planets. Extravehicular activities (EVAs) conducted by astronauts on Mars will require the astronauts to be more flexible and adaptive and to adopt a larger degree of autonomy than in EVAs conducted to date, due to the working environment introduced by non-negligible communication latency and bandwidth limitations between Mars and Earth. While astronauts on Mars will operate more independently than Apollo astronauts, they will still be expected to receive guidance from a team of experts on Earth gathered into a Mission Support Center (MSC) (Eppler *et al.,* 2013; Yingst *et al.,* 2013) due to the limitations of training future astronauts in the sheer breadth and depth of science that will be required for effective astrobiological and geological exploration.

An effectively organized and trained MSC can support astronauts with tactical decision-making during an EVA to guide them toward the best possible sampling decisions, as long as the EVAs are designed to consider this input in relation to the latency experienced between the crew and the MSC. Various setups and communication schemes between a terrestrial MSC and extravehicular astronauts have been developed and tested during previous real and analog planetary exploration missions (*e.g.,* Eppler, 2011; Lim *et al.,* 2011), but the communication latency between any teams on Earth and Mars presents a significant challenge to effective communication and decision-making. This challenge is only enhanced by the uniquely complicated task of astronauts performing aseptic sampling in environments more extreme than any on Earth. Searching for and selecting the samples that could hold evidence for life requires tactical planning and well-established protocols between the crew on Mars and the MSC, given that the time available to actually conduct science may be relatively limited in the timeline of the entire surface mission (Eppler, 2011). The process of collecting samples with astrobiological implications will also be more time intensive than sample collection during Apollo; and when combined with restrictions on both forward contamination (for planetary protection reasons) and cross-contamination in order to reduce scientific uncertainty (Rummel, 2001; Rummel *et al.,* 2014; Kminek and Rummel, 2015), as well as the technological limitations of *in situ* analysis and mass restrictions of sample return, the crew will need to be judicious in their approach for sample selection. Previous analog investigations have tended to (understandably) focus primarily on science questions and the technical methods used to answer those questions, but relatively few have focused on the operational and human factors that influence the decisions that must be made in this process (*e.g.,* Lim *et al.,* 2010; Thiel *et al.,* 2011). However, more work is this arena is required, since the standard protocol that terrestrial field science generally follows might not be most appropriate or effective for application to extraterrestrial exploration missions, and should be critically appraised and developed before humans are sent to other planets.

During the NASA Biologic Analog Science Associated with Lava Terrains (BASALT) research program, we conducted scientific research under crewed mission simulations, including an expected minimum and maximum communication latency between Earth and Mars. Here, we describe how tactical decisions were made in concert by the “terrestrial” MSC and the “martian” crew during two BASALT deployments to Mars analog field sites. Tactical decisions are those made within the context of a single EVA, whereas strategic decisions are made before and between a series of EVAs. Examining the tactical decision-making in the BASALT simulated EVAs provides input into how best to plan future astrobiological sampling EVAs in crewed missions to Mars.

## 2. Background

Current plans for Mars exploration involve robotic precursors (Fong *et al.,* 2010) followed by human exploration (Drake *et al.,* 2010; National Research Council, 2012). As the question of life on Mars is one of the highest priorities for space agencies around the world (International Space Exploration Coordination Group, 2013), it is inevitable that these crewed surface missions will involve some astrobiological sampling.

An astrobiological (rather than purely geological, as in the Apollo missions) focus will place a number of scientific requirements on a crewed Mars mission. Astronauts will have to collect a suite of samples representative of a given location to ensure scientific reliability and to account for the limitations of biological analyses, using techniques that do not degrade or damage potentially important biosignatures. They will have to apply a range of life-detection techniques (that may not exist yet) to the samples, whether *in situ*, in a lab located in a Mars base, or on Earth after sample return, and allow the information from these samples to be combined with environmental information, such as contextual geochemistry, to give a full understanding of the past or present ecology of the sampling site. The mission will be limited in the number of samples that can be returned to Earth for detailed laboratory analysis due to mass restrictions (Moores *et al.,* 2012), so each sample suite will have to be chosen carefully and possibly down-selected from the full range collected.

In addition to the astrobiological sampling requirements, the astronauts will have to deal with contamination issues. The most likely places on Mars (by our current understanding) to harbor life are currently defined as *Special Regions* because they are also most likely to be contaminated, interfering with any extant ecosystem and potentially “spoiling” science return (Rummel *et al.,* 2014). This means that the methodologies for these crewed missions must also aim to reduce the possibility of forward and cross contamination.

The requirements and limitations placed on these future crewed surface missions will create a unique work environment for the astronauts, one that we can plan and test protocols for in advance using analog missions. One aspect of the BASALT program was to identify how to best meet astrobiological sampling requirements while maintaining adequate contamination control measures and impacting as little as possible on science return.

When compared to other exploration contexts, the context of advanced planetary field geology and biology sampling places particular importance on real-time adaptive planning, high levels of geological expertise, and advanced tools and *in situ* analysis (Hodges and Schmitt, 2011; Schmitt *et al.,* 2011). A common work environment with multiple operators requires constant cycles of communication, action, and feedback (Hollnagel, 1998), and previous studies have identified prior scientific training of nonspecialists as a critical component in the scientific success of simulated EVAs (Love and Bleacher, 2013; Yingst *et al.,* 2013).

In this manuscript, we discuss how tactical decisions can be made collaboratively between crew on “Mars” in concert with a MSC on Earth as a means to ensure desired science return. We define *tactical decisions* as those made within a planned EVA, where the time horizon goes beyond present needs but is limited to within the EVA (Hollnagel, 1998), under the time constraints associated with round-trip communications latencies and other operational restrictions representative of Mars surface operations (Beaton *et al.,* 2019a). An overview of the structure of the BASALT program, which included such modules as astronaut training, communications infrastructure and software development, operational testing, and science, is described in Lim *et al.* (2019). The scientific context for our objectives is described in Hughes *et al.* (2019), and more detail of the strategic planning and decision-making is described in Brady *et al.* (2019). The layout and functioning of the MSC, as well as how it was adapted in response to changing needs, is described in Payler *et al.* (2019), and the concepts of operations (ConOps) for how the EVAs were run are described in Beaton *et al.* (2019a). Here we examine the influence of the conditions imposed by the mission architecture on scientific decision-making at different stages during an EVA. We look at how this decision-making process responded to the requirements placed on it by sterile sampling, mission flight rules, communications latency, and bandwidth limitations to achieve meaningful geobiological sampling under simulated mission constraints. We identify key insights from our simulations that aim to inform future mission planning from the perspective of tactical scientific decision-making.

## 3. Methods

The operational and decision-making structure used by the BASALT program has been developed over time from a range of experiences including the Apollo missions, various Mars rover missions, and a number of analog studies in terrestrial and aquatic environments (Clark, 2010; Lim *et al.,* 2011; Bleacher *et al.,* 2013; Eppler *et al.,* 2013; Yingst *et al.,* 2013; Beaton *et al.,* 2017). While top-level scientific priorities are made by the Mars-based crew and Earth-based support personnel in concert during the planning stages, the communication latency introduced in a Mars mission dramatically shifts the decision-making process during EVA execution from what has previously been used in terrestrial, lunar, or robotic Mars missions. The complete operational structure is described in detail elsewhere (Beaton *et al.,* 2019a) but is summarized here where it relates to decision-making. Personnel in our team ranged from undergraduate students to astronauts with in-orbit experience but together formed a group of around 70 people with hundreds of years of experience in disciplines critical to the BASALT program, such as space operations, communications and data networks, geology, and biology. More details of the personnel forming the EV, IV, and MSC teams and their respective interactions are given in the works of Beaton *et al.* (2019a) and Payler *et al.* (2019).

The two main scientific decision-making “units” during BASALT EVAs were the Extra-Vehicular (EV) and Intra-Vehicular (IV) crew as one unit in communication in real time (EV/IV), and the MSC. Each EV and IV pairing was formed of one operationally focused crew member (EV1 & IV1) and one scientifically focused crew member (EV2 & IV2), each with particular responsibilities. These pairs of crew members remained consistent through a deployment and rotated between positions in EV, IV, and MSC. This allowed the EV/IV pairs to develop a partnership and ensured they had insight into how each team worked and communicated. During our BASALT simulated EVAs, EV/IV crew and the MSC were separated in space and, depending on the particular simulation parameters, varying degrees of time, in addition to having limitations on data transmission rates between them.

The low and high latency conditions investigated in BASALT operations are 5 and 15 min one-way light time (OWLT) communication latency, which fall within the 4–22 min OWLT delays experienced between Earth and Mars due to orbital alignment. The bandwidth transmission restrictions are a conservative case of 0.512 Mb/s uplink and 1.54 Mb/s downlink, representing low-cost communications infrastructure, and a high bandwidth case of 5.0 Mb/s uplink and 10.0 Mb/s downlink, representing abilities upgraded from current infrastructure. Full details of the reasoning behind the BASALT ConOps is given in the work of Beaton *et al.* (2019a).

Each EVA had a set of clearly outlined science objectives that were determined at a strategic level prior to EVA execution (Brady *et al.,* 2019). Science objectives in each EVA were for EV crew to find and sample particular types of altered basalt, returning with one or two sample suites used to investigate the relationship between geochemistry and microbial habitability in basaltic environments. More detailed scientific questions were developed for the program (Lim *et al.,* 2019), but during an EVA the top-level question was divided into specific targets for the crew to identify. Having nonsimulated scientific objectives for the fieldwork that formed our simulated missions meant that we could more accurately determine how these scientific questions were affected by the operational limitations imposed by the mission protocols.

Overlaid on our science objectives were a set of defined procedures and flight rules, which maintained consistency between EVAs. These procedures told each crew member what they needed to do at each stage of the EVA, and the flight rules determined operational limits such as “hard stop” and “no-go” conditions (Beaton *et al.,* 2017).

Under communication latency, the sequencing of communications between two parties becomes important to avoid confusion (Love and Reagan, 2013), as otherwise the two parts of a conversation will arrive out of sequence. Previous analog operations have found that this sequencing becomes untenable with voice communications between the MSC and EV crew members, hence the MSC communicated with IV crew members across the time delay using a text-based system, whereas EV and IV crew remained in real-time voice communication. The MSC heard all the communication between the EV and IV crew members, and IV could, if desired, direct voice communication to the MSC; but the MSC did not communicate with the EV crew via voice. Since precise and consistent voice communications that use pre-established terms are important, especially in a low-bandwidth condition where interference or lost packets can disrupt communications (Love and Bleacher, 2013), all EV/IV crew were trained in appropriate radio language using a protocol document assembled by the operations researchers based on previous programs and missions.

The BASALT research program used a number of tools to enhance our communications and collaboration between the EV/IV crew and MSC and assist with decision-making. The *Minerva* software platform, developed for and during the BASALT research program, was created from software used in a number of prior analog missions, incorporating three components—Playbook, xGDS, and SEXTANT. These components were used in various strategic and tactical planning contexts throughout the BASALT program. Playbook is a scheduling and real-time execution tool as well as a text communication interface (Mission Log) that accounts for OWLT latency between users in different locations and is described in detail in the work of Marquez *et al.* (2019). The Playbook Mission Log (which also automatically logs all the messages) is the main method of communication between the MSC and the EV/IV crew. xGDS (eXploration Ground Data Systems) is a combined set of tools for mapping; traverse planning; real-time visualization of EV crew location, activity, telemetry, and scientific data; note taking; and includes a data archive for later analysis (Deans *et al.,* 2017). xGDS is the main scientific data system used by the MSC, as it shows them the location of the EV crew on a map (which can include multiple geographical data layers), displays the real-time video (if available), and catalogs the still images and instrument data sent back by the EV crew. SEXTANT is a path planning tool that aims to optimize traverse over terrain using digital elevation models and cost functions. While SEXTANT was mainly used for traverse planning in the BASALT research program (Brady *et al.,* 2019), it has the capability of real-time adaptive replanning that could be used within an EVA.

**FIG. 1.**
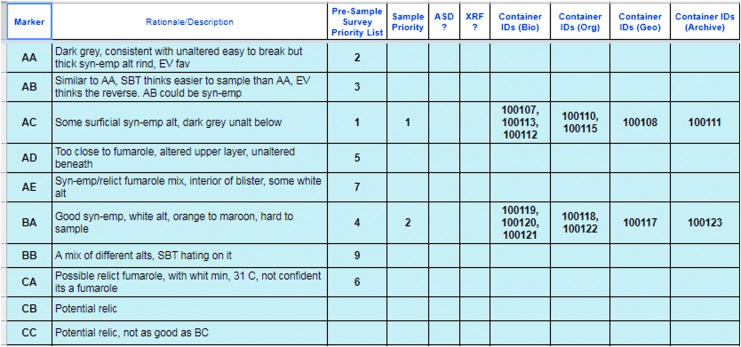
Example screenshot of an EVA leaderboard master sheet, filled in by the personnel assigned to Leaderboard in the MSC. For each candidate marker placed by the EV crew, a summary of the science discussion in the MSC was included and the candidate given a priority for the second and third EVA phases. Note that not all candidates were given a priority, if they were deemed not to meet the EVA's science objectives. If samples were collected from a candidate, the container numbers were included for archival purposes. The full MSC does not see this version of the leaderboard, but examples are shown in Fig. 2 or Fig. 3 depending on the phase of the EVA. The marker labels were AA, AB, AC, and so on for the first station and BA, BB, … and CA, CB, … for the second and third station, respectively. As the leaderboard was prepared under time pressure, slang was often used. SBT = Science Backroom Team, an alternative name for the Science Support Team.

**FIG. 2.**
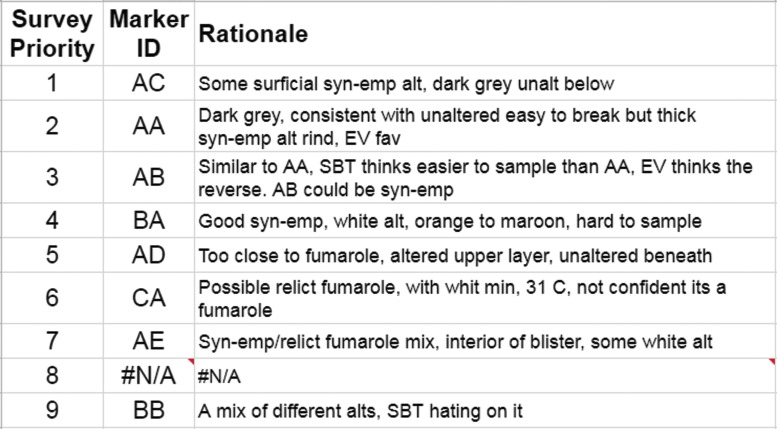
An example of a presampling survey leaderboard derived from the backend shown in Fig. 1. Note no candidate at position 8, due to the MSC placing very low priority on Candidate BB.

**FIG. 3.**
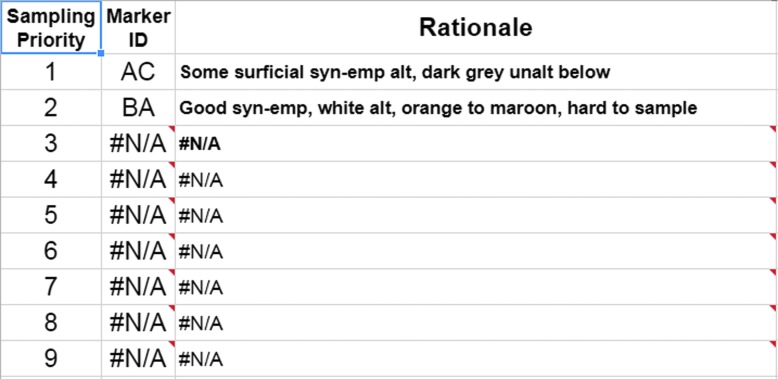
An example of a final sampling leaderboard derived from the backend shown in Fig. 1. Note only two candidates were included after the presampling survey.

BASALT also incorporated compact field instruments with real-time data return, which allowed the EV crew to complete *in situ* analyses and send the results back to the MSC (Sehlke *et al.,* 2019). The information provided by these instruments was used in the presampling survey phase of our EVAs to down-select the candidate sample locations before final sampling. Down-selection was facilitated by using a dynamic leaderboard created using a spreadsheet (Figs. 1–3), which allowed the MSC to place the candidates in a priority order depending on the information delivered by the EV crew at various periods throughout EVA execution. The leaderboard represented the MSC preferences on a moment-by-moment basis based on the latest information provided by the EV/IV team. The MSC was frequently queried to ensure consensus was made among the various scientific experts particularly at key phases of the EVA.

The dynamic leaderboard formed an important part of the decision-making in the EVA and had a member of the MSC dedicated to updating and maintaining it (see Payler *et al.,* 2019). It allowed for the constant reprioritization of candidate locations without having to reassess all the previous candidates. Because only the highest-priority candidates needed to be transmitted to EV crew to allow them to continue the next phase of the EVA, time-critical decisions only required each new candidate to be assessed relatively against the top candidates in the leaderboard and a new priority list of 3–5 candidates sent. The leaderboard used in BASALT assisted in these decisions by including descriptions of the different candidate locations, reasoning for their priority related to the EVA objectives, and images of the candidate for quick reference.

**FIG. 4.**
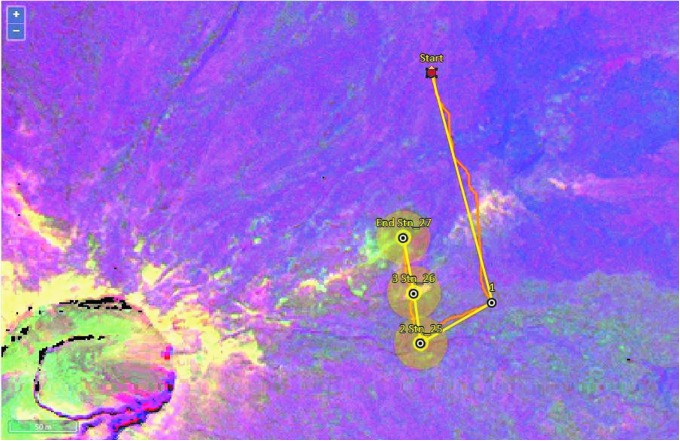
An example of a traverse plan created in the xGDS software, with local aerial photography and an overlaid (partially transparent) multispectral map. The EVA took place on Mauna Ulu (crater visible bottom left) and targeted potential alteration products highlighted by multispectral data. There were three stations, with one waypoint used to prevent cross-contamination of stations by guiding the EV crew to walk in from a particular direction that would bypass the other stations. The yellow line is the straight-line path between waypoints, and the orange line is the traverse path calculated by SEXTANT as the optimal walking path. Each station is an area with a diameter of 10 m (interior yellow circle) for the EV crew to explore and search for potential samples. The larger yellow circle around each station is provided to create buffer zones of increased caution regarding potential contamination of candidate samples, as well as extensions to the station area as necessary to meet the EVA objectives.

Extravehicular activity plans were established days, weeks, or even months in advance, using information from a number of sources, including aerial photographs and multispectral data (Brady *et al.,* 2019). An example traverse plan is shown in Fig. 4. Each EVA plan consisted of a short traverse and a selected number of stations for the astronauts to visit and collect sample suites from.

**FIG. 5.**
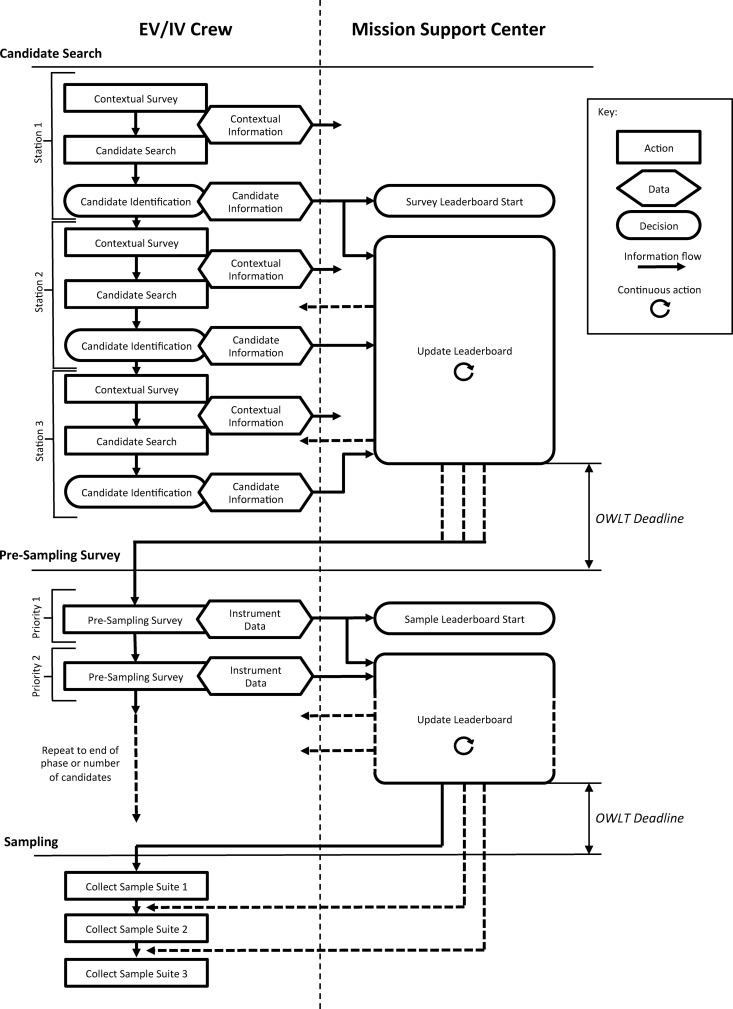
A schematic view of the EVA timeline and the decision-making within it. Decisions are initially made by the EV/IV crew, choosing candidates and sending them to the MSC. When they receive candidate information, the MSC begins creating and updating leaderboards, sending them to EV/IV as they progress, which continues until a deadline set by the OWLT (5 or 15 min) for the experimental condition, at which point they must have sent at least one version of the leaderboard to allow EV/IV to move on to the next phase of the EVA. During the presampling survey, the EV/IV decision-making is much more limited, and their tasks mainly consist of sending more detailed information to the MSC to allow them to choose which candidates should be sampled. The leaderboards in each phase are continuously updated in response to new information and can be sent many times, as long as the EV/IV crew have at least one priority to work from at the beginning of the next section.

**FIG. 6.**
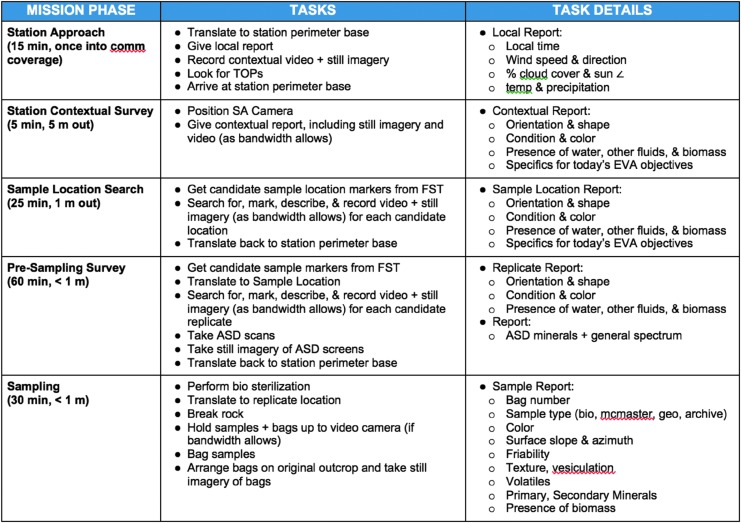
Example cuff checklist giving the EV crew direction on what they should be doing at each stage of the EVA. TOPs = Targets of Opportunity, SA = Situational Awareness, FST = Field Support Team, ASD = Handheld VNIR spectrometer (later supplemented by a portable XRF unit).

Extravehicular activities were organized into discrete activities arranged in a number of phases (Figs. 5 and 6). Activities were assigned blocks in the planned timelines. A detailed breakdown of EVA structure and tasks is given in the work of Beaton *et al.* (2019a).

In each of these phases, decision-making operated differently, as summarized schematically in Fig. 4 and in list form in Table 1. EVAs began with a recon phase in which the EV crew moved between three planned stations and searched for candidates as defined by the scientific objective for the EVA. During this search, the EV crew provided information using vocal descriptions, photographs, and video (where bandwidth allowed) and proposed sample locations by placing markers with alphabetical codes. The MSC used the contextual information provided to down-select the candidate locations proposed by the EV crew and assemble a leaderboard (Payler *et al.,* 2019).

**Table 1.** Timeline of a BASALT EVA, Showing the Timing of EV/IV and MSC Activities under Different Communication Latency and Allowing for Exploration of Three Stations in One Site

In the second phase, the EV crew return to candidate locations they have identified and use stand-off and contact instrumentation, including portable X-ray fluorescence (XRF) and visible and near-infrared (VNIR) spectroscopy, to provide mineralogical and geochemical information about the candidate sample locations (Sehlke *et al.,* 2019). Critically, in both of these phases the EV crew aimed to avoid contamination where possible and limit their travel around the vicinity to reduce cross-contamination, with a mind to wind direction (as identified in the contextual survey at the beginning of the EVA). They did use rock hammers and other tools to explore the general geology of the site but avoided contact with any potential sampling location. This is in contrast to Apollo EVAs or prior analog deployments that were not astrobiology-focused, where crewmembers could explore the site in as much depth as they wanted before sampling, as contamination was not a factor.

After the MSC prioritized the candidate sample locations for the final phase using verbal descriptions, contextual photographs, and instrument data, the EVA moved into the sampling phase. At this point, decision-making in the MSC was limited and became more about the crew on the ground deciding how best to extract a particular sample. While the MSC could make limited contributions that were highly dependent on the latency condition and rate that the EV crew worked at, this phase relied heavily on the prior training of the EV crew in sterile sampling methods.

Each EVA phase is sequenced in some part to ensure that EV crew downtime is minimized. For example, after identifying and describing candidate locations at the first station, the crew moves to the next station and repeats the candidate location search in the new area. MSC processes the information from the first station and prioritizes the candidate locations while EV are at the second station, meaning that even under the 15 min latency condition, the EV crew receive the location priority information before moving back to this location; thus, the EV crew are not left waiting for information, wasting resources and consumables on the martian surface. This is in contrast to rover mission ConOps (*e.g.,* Moores *et al.,* 2012), which may include periods of idle time during data processing and interpretation. In the case of a rover, this idle time is undesirable though perhaps necessary when power-limited, as the rover must have regular periods of idleness when using solar panels to recharge. In the case of a crewed mission, EV crew idle time means wastage of mission-critical consumables. In general, we aimed to avoid EV crew waiting for a response from the MSC by having clearly defined “bingo times” that identified the latest time in which particular information must be sent by the MSC to keep the EV crew on schedule. These bingo times were monitored and managed by the SciCom position within the MSC and by the IV crew (Payler *et al.,* 2019).

In the early phases of the EVA, the MSC made decisions in a framework established by the information provided to them by the EV/IV crew, whereas in the later phases of the EVA the EV/IV crew made decisions within the framework of priorities (and reasoning) provided to them by the MSC. We will examine these in turn.

### 3.1. Phase 1—Site recon and candidate search

**FIG. 7.**
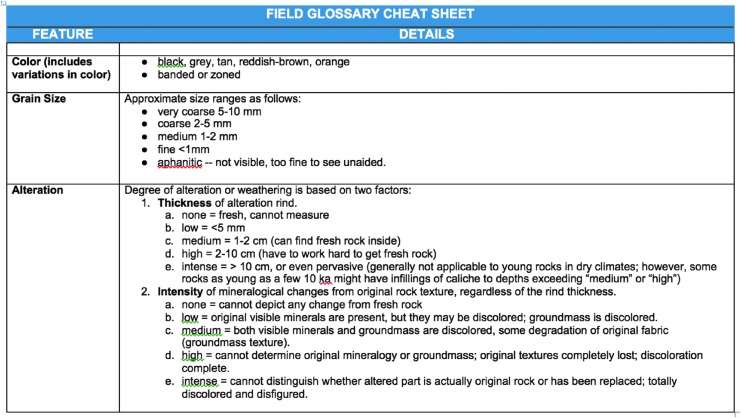
Example cuff checklist giving the EV crew information on what they should describe about the candidate sample locations.

In the initial phase of the EVA, the EV crew navigated to a station waypoint, as defined in the EVA plan, using GPS and directions from the IV crew. Once at a station waypoint, the EV crew members provided a contextual survey of the area around the station using a combination of verbal descriptions, video footage, and photographs at varying resolution including orientation and scale markers. Figures 6 and 7 give examples of the cuff checklists used by the EV crew as prompts for what descriptions they should be making at different stages in the EVA. The video from the EV crew cameras was sent at 720p resolution but displayed on screens in the MSC at a downscaled resolution around half this.

**FIG. 8.**
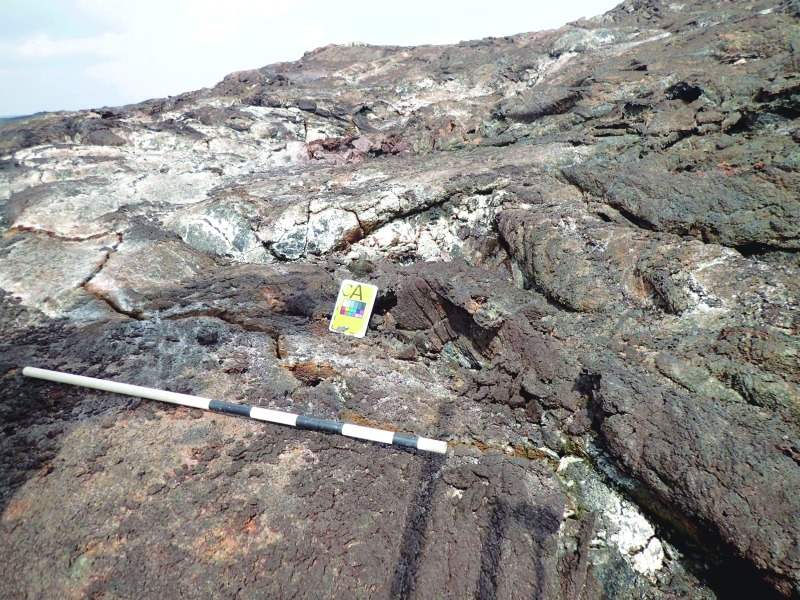
An example of a context image of a sample marker laid by EV crew, identifying a potential fumarole for later sampling. EVA procedures called for an initial context image with a scale bar, followed by close-up shots of the proposed sample location. Note the marker and scale bar are laid away from the location intended for sampling (the crack between the “CA” marker and the scale bar) to prevent contamination. In some cases it was necessary to make the intended target clearer by pointing with improvised devices, but this information was mostly relayed over voice communication and recorded by stenographers in the MSC.

**FIG. 9.**
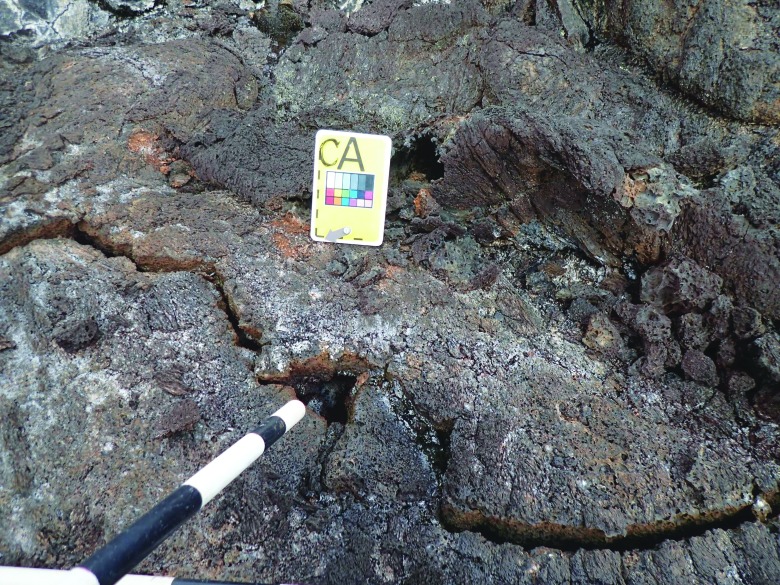
An example of a close-up shot of the same sample marker as Fig. 8, laid by EV crew to identify a candidate fumarole location for sampling. EV made the target clear to MSC in this case by pointing with a scale bar.

After giving a general context for the station, the EV crew systematically explored the area and looked for candidate sample locations that met the specific criteria as established in the BASALT Science Matrix (Brady *et al.,* 2019). Whenever they identified a candidate, they placed a unique marker and sent extensive verbal descriptions and still images of the proposed sample location (see Figs. 8 and 9 for examples). Generally, one context image and one close-up image were sent for each candidate, but this was not limited, and sometimes the EV crew decided to take three or four. These images were taken at 8 megapixel resolution in the high-bandwidth condition and at 8 and 3 megapixel resolution in the low-bandwidth condition, with only the 3 megapixel images available to the MSC. For our science objectives, which were differentiated by different types and gradings of basaltic alteration, critical descriptions and imaging focused on the colors and textures of the alteration in and around the candidate locations. By comparing the descriptions made by EV crew and the images they sent back, the MSC decided (a) if a candidate matched one of the alteration conditions required by the science objectives and (b) if it was a better or worse example of this alteration condition than what had been previously found by the EV crew.

This phase of the EVA (defined as contextual survey and sample location search) was limited to 30 min per station but was not limited in the number of markers that the crew could put down. After the allotted 30 min, the crew would move to the next station and repeat the process. This limit was thought to be enough time to make an initial exploration of the area while enabling the crew to cover multiple potential locations within to the total time limit of the EVA. The limit could be overridden by the scientist EV crew member if they deemed the station important enough to warrant it and the additional time did not break any of the flight rules.

This first phase is therefore dominated by decisions made primarily by the EV crew, with support from the IV crew, who have real-time access to the video and photographs relayed by the EV crew. While the stations are predetermined in planning, the crew has autonomy in choosing the possible sample locations to mark within the stations. Their choices were based on their own training and prior field experience, directed by the scientific objectives of the EVA. For each candidate sample marker, EV2 would provide a verbal description of the marked location, including a statement of what EVA science objective this possible sample location would meet. EV1 would provide context and close-up photos of the marked location with scale bars (1 m staff with smaller increments marked with tape). Three to six candidate locations were chosen per station. This collection of information (voice descriptions, notes from IV crew, and images) was transmitted back to the MSC (and viewed in xGDS), where they discussed and ranked each candidate location.

A predetermined imaging protocol gave the EV crew guidance for how to take images at each phase of the EVA. Each station began with four images in the four cardinal directions, with scale bars in the foreground, to provide a general context for the station. This also gave a visual cue to the MSC that a new station was being explored. After this, each station typically involved several overall contextual images and three to five candidate locations (the maximum was six, but the number was not constrained by anything other than time), each with at least two images—context (Fig. 8) and close-up (Fig. 9). This collection of images and description formed the parameter space of information that the MSC then used to rank the candidate locations. Typically the close-up images were the most useful information for the MSC, as they were generally able to discriminate different alteration types by eye. However, in many cases the MSC relied on the EV's own scientific interpretation of the location and used their confidence to decide on a priority order. The thoughts and recommendations of the EV crew were captured by stenographers in the MSC, as these could easily be lost among the MSC discussions. Following the sequencing of the imaging protocol was critical, as the information in each image had to be tied semantically to the verbal descriptions provided by the EV crew.

As soon as information on the first candidate location was received by the MSC across the OWLT delay, the MSC began assessing and prioritizing the information on a dynamic leaderboard to identify which candidate locations should receive further scrutiny using the scientific instruments carried by the EV crew during the second phase of the EVA (presampling survey). The leaderboard was updated as data for each candidate sample was received and assessed, and the current list was transmitted back to IV crew through Playbook with short notes on the MSC's rationale for the order. The MSC had a time-critical deadline (bingo time) to respond to the IV crew with a prioritized leaderboard of as many of the candidate samples as possible before the EV crew finished exploring the third station; remaining candidate samples from the third station that had not been integrated into the leaderboard were continually added as the EV crew progressed through the presampling survey phase. The limit placed by the OWLT on the first leaderboard response was the first critical decision-making point in the EVA, as without at least one priority recommendation the EV crew could not move on to the next phase, so the leaderboard had to be sent at least 5 or 15 min in advance. Synthesizing the recommendations of the different members of the MSC was assigned to the two Biology and Geology Leads, who each reported a final recommendation to the Science Lead. The recommendations were most often in accordance, but in some cases there was a difference, highlighting the need for the MSC Lead to be a scientist by training. The Science lead used these recommendations to make a final decision on the leaderboard prioritization, which was then sent to the IV crew (Payler *et al.,* 2019).

From the perspective of the EV crew, the exploration of the three stations was followed sequentially by receiving the candidate leaderboard and moving on to the next phase. Conversely, from the perspective of the MSC, these events were occurring in parallel—they had to send a leaderboard before the EV crew finished at the first station, and while EV were working at the next station the MSC was still looking at information from the previous one. This meant that the MSC was continuously reprioritizing the leaderboard in response to new information received from the EV crew. After the initial, time-critical transmission of the leaderboard, the pressure on the MSC was relieved slightly, and they had some time to continue science-focused discussions and reprioritize the entire table. In some cases, the leaderboard was completely reordered in response to these discussions.

Because of the structure of the planned EVAs, there was a subtlety to the initial prioritization in the dynamic leaderboard. During the candidate sample location search, when the dynamic leaderboard for the upcoming presampling survey was being built, the highest-priority candidate was not necessarily what the MSC thought was the highest sampling priority, but rather the candidate that they most wanted scanned with the scientific instruments in order to enable better decisions on the eventual sampling priority. For example, where one candidate had been identified for the primary science objective, but several candidates had been identified for the secondary science objective, the MSC prioritized the multiple secondary candidates for instrument scanning to allow these to be ordered appropriately beneath the primary candidate. In this case, the top priority in the presampling survey leaderboard would not be the same as the top priority in the sampling leaderboard.

### 3.2. Phase 2—Presampling survey

Before the beginning of the presampling phase, the EV/IV crew should have received a prioritized list of locations to move back to and assess in more detail using scientific instruments. In our EVAs, this target was always met; but if it had not been, the EV/IV protocol was to begin working on their own initial priority rather than waiting for input. Depending on the specific context of the EVA, the highest priority could be from the first or second station, or in the case of a 5 min OWLT, the third station as well. At this stage in the EVA, the EV crew had an extensive number of tasks to accomplish but few decisions to make. They worked autonomously within the list of candidates given to them by the MSC to provide more information, with some decisions about how best to achieve this, but only in a minor sense. They could choose exactly where in an outcrop to sample but not which outcrop to sample. The EV crew were also given some autonomy in determining the order in which they would visit the candidate sample sites. For example, in some cases they could visit candidates at Station 2 first because it was closer to their current position. In some cases the MSC felt they lacked some information and deferred to the EV to use their best judgment as to which of two sites was a better example.

The EV crew used handheld instruments to scan representative surfaces at the candidate locations and transmitted the resulting data, as well as additional photographs (which were typically close-ups) and observations, back to the MSC. This phase in the EVA was less structured in the timeline, without specific intervals assigned for the three separate stations, as the leaderboard could have candidate locations from any of the three stations in any order. However, there was again a critical cutoff time where the MSC needed to provide an updated leaderboard to EV/IV in order for them to move to the sampling phase. In many ways this phase of the EVA was similar to the first. Working within a predetermined plan, the EV crew transmitted information to the MSC that allows them to down-select candidates for the next phase. The MSC perceives these actions to be happening in parallel, whereas the EV crew perceive them sequentially, and the IV crew experiences a hybrid of the two, as they are managing the information flow between MSC and EV.

The aim of the second phase was to critically refine the priority listing on the leaderboard based on additional information offered by the handheld instruments. This meant the decision-making was slightly different in the two phases. In Phase 1, decisions needed to be made about whether candidates met the science objectives for the EVA, which of the candidates appeared to be better examples of the conditions described by the science objectives, but also which of the candidates required further investigation to answer these questions. In Phase 2, the only decisions were which of the candidates were the best matches to the science objectives, but additional information was provided to the MSC.

### 3.3. Phase 3—Sampling

The final phase of the EVA involved relatively little decision-making on the part of the MSC. Once the candidate samples had been prioritized and the sampling leaderboard transmitted back to EV/IV, there was little to do except sample. Theoretically it would be possible for the MSC to change the second-priority sample location while the EV crew was sampling the first priority, perhaps if they continued analyzing photographs and data and changed their mind, but in practice this did not happen.

**FIG. 10.**
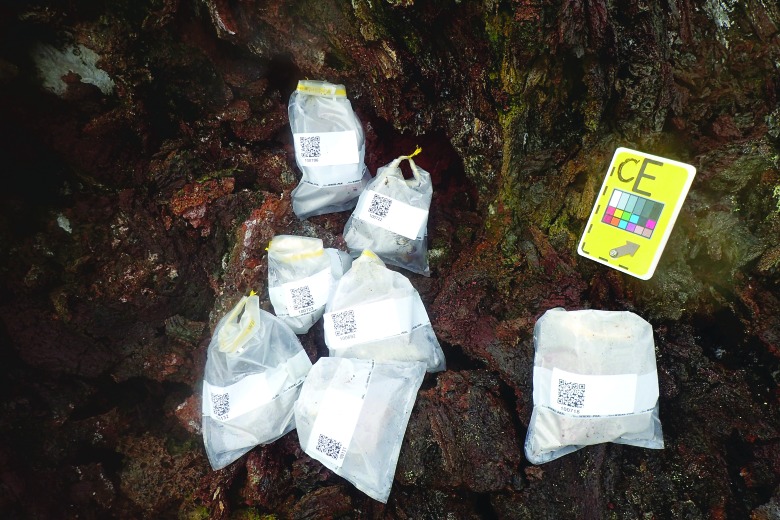
A suite of samples collected from a “CE” candidate location. Each set of sampling ended with a similar photograph of sample bags *in situ* to provide context and specific localization.

Despite the majority of the decision-making being complete, there was still work for the MSC to do in the final phase. EV crew continued transmitting verbal descriptions and photographs, providing the MSC with further information that they could use to interpret and assess the samples being taken. In addition, there was a significant amount of “book-keeping” work such as completing notes, tagging photographs, and matching sample numbers to sample bags (see Fig. 10).

## 4. Results and Analysis

A number of metrics were collected on all aspects of the BASALT research program. These will be discussed in depth elsewhere (Beaton *et al.,* 2019b); here we focus on the qualitative results applicable to tactical decision-making.

### 4.1. Crew and MSC roles

The liaison roles (Biology Lead and Geology Lead feeding to Science Lead, and the SciCom who communicates between Science Lead and IV) between EV/IV and MSC proved to be critical in enabling efficient decision-making, as the communication between the two groups was critical. Information flow was mainly one way, from EV/IV to MSC, but there is important information that the MSC must send to EV/IV in a clear and efficient way, via text communication through Playbook. With a large MSC, it was important to have liaison roles staffed by those able to consolidate the group decision-making, emphasize time-critical cut-offs, and send the digested information to EV/IV. Our formal MSC structure (Payler *et al.,* 2019) had two science subleads (Geology and Biology) who digested information from a subset of the MSC and deferred to one overall Science Lead, who could then consolidate this information and break any deadlocks that might have happened between the two teams. The particular techniques of the Science Lead role depended heavily on the personality of the person in the role, as these were rotated—some sent far more but often shorter messages than others, whereas some tended to use a “digest” format sent less often. There was not an appreciable difference in outcome between the different styles of Science Lead (Payler *et al.,* 2019).

Another MSC role that proved to be critical was that of the Stenographer, and in fact having multiple independent Stenographers listening to the voice channels of the EV crew. Because the MSC was continuously receiving new information while also discussing this information and making decisions, different members of the MSC often missed important incoming information from the EV and IV crew while the MSC members worked together to assess data that had been previously received. Having several appropriately trained members of the MSC focused on the voice discussions between EV and IV meant they could alert the rest of the team to any important information that might have been missed during discussions at a time that was most appropriate for the MSC (*e.g.,* after a given discussion about a past set of information was completed).

The importance of scientific training was also incredibly important for the EV crew. Because the MSC can only make decisions on information provided by the EV crew, that information must be of high quality. Verbal descriptions needed to be clear and concise but carry enough detail. Photographs needed to be appropriately framed and scaled. Having all members of the EV and IV crew well trained in what to look for when identifying candidates meant information flow was not dependent on the single “scientist” EV crew member, as both of the EV crew could cover different areas of the stations (in the search phase), but also that EV/IV could engage in useful real-time discussions that often helped resolve a scientific question without having to defer to the MSC and wait for a response. It also meant that the EV crew could have input, which was prompted for by the IV crew as part of the EVA protocol, into the eventual ranking in the leaderboard or choosing between two equally ranked candidates, which proved to be very useful when there were unclear or borderline candidates, and the MSC placed a high weight on the opinions of the EV crew where given.

### 4.2. Capabilities

A number of technological capabilities were identified as being important to the decision-making process. The BASALT research program uses xGDS software as its main tool (Marquez *et al.,* 2019), which allows for EVA planning and consolidation of text, video, photographic and instrument data. A tool like this, which has been developed over a number of analog projects, proved to be invaluable for enabling the MSC to make decisions, given the large number of data streams they must evaluate information from. Being able to quickly switch between images annotated with metadata and glance at real-time video from the field allowed the scientists in the MSC to essentially “inhabit” the same space as the EV crew, enabling a much deeper scientific understanding of the sampling context. Another aspect of the system that proved to be useful was the ability to annotate images from the field to send back to EV/IV with specific directions. In cases where the MSC wanted to be very specific about what they wanted to sample, verbal descriptions were not adequate to pass on the required information. However, as these cases were relatively rare, it was not possible to accurately assess how critical this capability was.

The planned timeline and procedures for BASALT EVAs required constant communications, even though they were subject to latency. Procedures and flight rules were included that allowed EV and IV to react to a loss of communications with the MSC or between each other, which did happen on a few occasions, but most of the time these reactive procedures did not affect the scientific decision-making as this was reduced in priority compared to safety in these cases. In a real Mars mission, where infrastructure such as relay orbiters will most likely be restricted, communications might be regular but interrupted, and the BASALT timeline concept would need to be adapted. Restricted twice-a-day communication downlinks would not easily be catered for under our operations concept, and the MSC would be far less able to influence science operations. In fact, MSC members said that hearing the thought processes of EV and IV was absolutely critical for understanding the context information they provided, which would not be possible in twice-a-day communications (Bleacher *et al.,* 2013).

### 4.3. Effect of different study conditions

Our study conditions involved low and high latency cases and low and high bandwidth cases. Each of these cases brought its own challenges and learning points. The EVA timeline in our study was designed to accommodate the high latency case, so for example the sample location search at each station was planned to take 30 min, allowing approximately for the 15 min OWLT transmission from EV/IV and response from MSC. This schedule assisted the MSC in making decisions on the scientific information, as they had time to process it before the next phase (with each phase generally involving an activity three times), though this relied on the MSC being provided sufficient information by the EV crew.

However, there was an interesting juxtaposition of the perception of how much the MSC could influence the EVA activities and the time pressures introduced by the communication latency. In the 5 min OWLT low latency case, where the critical time cut-offs for MSC decision-making were 10 min later than in the 15 min OWLT high latency case, the decisions in MSC were generally more “frantic” and pressured in an attempt to influence the EVA more directly. This is counterintuitive but seemed related to the fact that the MSC felt better connected to the EV crew under low latency, whereas under high latency there was less opportunity to directly influence the EV crew. In the high latency case, where critical time cut-offs were earlier, there seemed to be a more “measured” approach as the MSC knew that it was more limited in how much it could influence the EVA. The more measured and relaxed approach meant that outside of the critical bingo times, the MSC waited for more information and was more methodical in analyzing it, while being mindful of the fact they had a deadline to make a decision. For example, in the high latency case, repeated updates of the leaderboard were often not sent as the MSC was aware they would have arrived too late to influence the next phase. However, in this case it was more important for the MSC to include precise reasoning for their priority list in the leaderboard, as it was more difficult for the IV crew to ask for clarification from them.

The main effect of the low bandwidth study case was that continuous video feeds were not transmitted to the MSC and the resolution of the photographs transmitted by EV crew was reduced (IV crew still retained access to the high-resolution imagery and video feeds). In general, the continuous video feed was a useful capability for the MSC as it allowed them quickly and simply to locate themselves with the EV crew and to understand the context of the sample locations. However, the lack of real-time video in the low bandwidth case was not severely detrimental and did not significantly alter the decision-making process, as multiple context photographs alongside extensive verbal descriptions were able to convey a large portion of this contextual information. The loss of high-resolution photographs (8 megapixel) in the MSC was not found to be detrimental, as the close-up, low-resolution (3 megapixel) versions were more than adequate for scientific interpretations of the geochemistry. Verbal descriptions and moderate-resolution images were more than sufficient for tactical decision-making.

### 4.4. Procedures

Although having EV crew sufficiently trained to recognize appropriate samples and make decisions about their quality proved to be an important consideration, we found that the EV crew often spent too long deciding whether a candidate was of a high-enough quality to place a candidate marker. While this was meaningful and important scientific decision-making, our ConOps placed this decision in the hands of the MSC. With MSC support, the EV crew should aim to place markers and move on, giving the MSC a wider choice to prioritize from rather than attempting to do this themselves. In the procedures developed for BASALT operations, EV crew made important decisions in what information to pass to the MSC; doing this in a systematic way that included important contextual details only evident to a well-trained crew member proved to be incredibly important. However, once they had collected that information, it was much more useful for them to place candidate markers at any potential sample location, rather than trying to threshold their quality during the EVA. This contrasts with the methodology used by astronauts in Apollo, where it was the astronauts that had the final say in whether to sample or not (Clark, 2010). In fact, there were some cases where discussions between the EV and IV crew about whether or not to place a candidate marker created confusion in the MSC. This confusion could be avoided by having a procedure that EV crew place a marker before they start discussing candidates.

Our experience also suggests that because EVA timelines were planned with the high OWLT latency case in mind, it was more beneficial for the EV crew to use up the allotted time in each activity rather than push to move to the next activity early. This meant where time gains were made, EV crew were better to push to find more candidate locations in the first phase, or perform more instrument scans of more representative samples in the second phase, than press ahead to the next station or candidate location. In general, sticking to the timeline allowed the MSC more time to process the information, especially in the high latency condition, reducing confusion, preventing the MSC from being overwhelmed, and keeping the two teams synced across latency. This is in contrast to previous analog missions without latency, where it has been found to be more useful to press to the next activity (Yingst *et al.,* 2013), but must be balanced with operational and safety concerns. Determining this balance was one of the goals of the BASALT program. In a real sequence of Mars surface EVAs over the course of years, the OWLT will change, and EVA timelines might have to be adapted; but in the case of a short-duration surface mission over the course of days, the time allotted to each activity could be planned with the specific OWLT in mind.

Overall, the procedures developed for the BASALT research program enabled high-quality scientific decisions. There were numerous examples where issues were identified with the procedures, and there were some occasions where communication breakdowns caused failures where decisions made in the MSC were not actioned correctly by the EV crew. However, to truly qualify whether our procedures improved scientific decisions over different procedures would require more extensive investigation, oversampling, and a method to critically assess the “quality” of samples, which was beyond the scope of this deployment. Nevertheless, the development of our procedures over the course of several deployments meant that while we did not completely achieve our scientific objectives in simulation during the first deployment (requiring additional, non-mission simulation sample collection), all scientific objectives were met in simulated EVAs in our second deployment.

### 4.5. Critical observations and information

In our study, the observations and information that were critical for decision-making were tied very closely to the science questions set at a program level. In general, the close-up images taken by the EV crew and sent to the MSC were the science data products most frequently relied upon to make decisions on priority in the MSC. These provided clear evidence of the different alteration conditions that informed our top-level science objectives. In some cases, data from handheld geochemical instruments or verbal descriptions supplemental to the images were used to confirm or refute the interpretation of the alteration conditions in the images, but rarely did they provide a completely different interpretation. However, this supplemental information was key when the MSC had to make a decision about which candidates to prioritize within the context of one specific science objective. Where some images were able to show us which rocks were “highly altered” or “unaltered” (for example), the detailed geochemical information provided by the instruments, and the strength of opinion of the EV crew, who had much a much better direct sense of the context and environment, were key in how the MSC prioritized the candidates.

In cases where the science objective of an EVA or the exploration in general is different, perhaps with a science question that is not as immediately visually obvious or one that is an example of a more binary than gradated condition, the balance of critical information might be different. This would suggest that when designing a generalized EVA for future Mars exploration, where the specific science objective is unknown and may vary significantly from instance to instance, it would be most beneficial to maintain the different streams of information available, as each stream might offer critical importance in different contexts.

## 5. Conclusions

A crewed astrobiology sampling Mars surface mission presents a unique context for scientific decision-making that has not been previously encountered by human exploration. The scientific objectives, contamination restrictions, communication latency, and bandwidth limitations all combine to form what will be a challenging activity for those people we send to explore the martian surface. A key point of the BASALT program has been to show explicitly that a MSC located on Earth can provide added value to astronauts performing astrobiological sampling on the surface of Mars, despite a communication latency and data transmission limitations between the two groups. With appropriate planning and timelining of activities and MSC staffing, a significant amount of decision-making can be offloaded from the crew on Mars to the MSC, which can contain far wider and deeper scientific knowledge and experience than the smaller EV and IV crew member team possibly can. Despite this fact, having crew members with a broad training in the appropriate geological and biological sciences proved to be critically important. Because the MSC can only make decisions within the scope of the information provided to them by the EV crew, it is important that the crew on the ground are able to identify interesting potential samples, explain why they are interesting, and understand how best to characterize them to pass on to the MSC.

Our simulations also demonstrated that there are a number of critical technological capabilities that need to be in place for such a mission. While constant video feeds from crew-mounted cameras proved useful, they rarely provided contextual information above and beyond what could be extracted from photographs taken by the crew, as long as those images followed the predetermined and systematic imaging protocol. However, uninterrupted voice communication was key in enabling the MSC to understand the reasoning of EV crew by listening to their thought processes and discussion with IV crew. Being able to send close-up photographs at reasonable resolution was the main route for scientific information to be passed from EV through IV and to MSC. It was also important that the photographs could be directed by the (highly scientifically trained) EV crew, rather than remotely operated for example, as they are necessarily in the best position to judge the best subject, contrast, and scale appropriately. With the development of more intuitive and user-friendly contact or stand-off geochemical instrumentation, the integration of these data products and their remote transmission may change the balance of information usefulness in future tests.

While other analog programs have investigated similar questions of decision-making and how science teams can operate in simulated extraterrestrial missions, there is little available literature detailing the background of particular protocols chosen, or examining in detail the results of these choices. We would encourage the community to publish more on these aspects of their investigations, as these discussions are clearly of interest as we move toward the human exploration of Mars.

## Abbreviations Used

BASALT: Biologic Analog Science Associated with Lava Terrains
ConOps: concepts of operations
EV: Extra-Vehicular
EVAs: extravehicular activities
IV: Intra-Vehicular
MSC: Mission Support Center
OWLT: one-way light time
VNIR: visible and near-infrared
XRF: X-ray fluorescence
